# Weigh change across adulthood is related to the presence of NAFLD: results from NHANES III

**DOI:** 10.1186/s12967-023-04007-8

**Published:** 2023-02-23

**Authors:** Lili Wang, Jiayi Yi, Jiajun Guo, Xiangpeng Ren

**Affiliations:** 1 Department of Biochemistry, Medical College, Jiaxing University, No.899 Guangqiong Road, Jiaxing, 314001 Zhejiang China; 2 Department of Cardiology, Fuwai Hospital, National Center for Cardiovascular Diseases, Chinese Academy of Medical Sciences and Peking Union Medical College, Beijing, 100035 China; 3 Department of Cardiology, West China Hospital, Sichuan University, Chengdu, 610041 Sichuan China

**Keywords:** NAFLD, Formerly obesity, Obesity duration, NHANES, Weight change

## Abstract

**Background:**

Obesity is a widely recognized driving factor of Non-alcoholic fatty liver disease (NAFLD), it remains unclear whether historical weight status was associated with the presence of NAFLD. The study aimed to explore the relationship between weight change across adulthood and the presence of NAFLD.

**Methods:**

Data from the National Health and Nutrition Examination Survey III included 6586 participants. Weight change was assessed according to body mass index (BMI) at baseline, at 25 years old, and 10 years before baseline. Obesity was defined as BMI ≥ 30 kg/m^2^. NAFLD was assessed by hepatic ultrasonography.

**Results:**

The prevalence of NAFLD was highest among stable obese participants (48.1%), and the lowest among stable non-obese participants (18.9%). Among non-obese participants, those who get obese in early adulthood had a higher risk for the presence of NAFLD than those who were never obese (odds ratio [OR], 1.82; 95% confidence interval [CI] 1.17–2.92). Among obese participants, those who become obese in middle-late adulthood had a lower risk of NAFLD (OR, 0.79; 95% CI 0.65–0.96) than those with stable obesity. A weight gain of more than 12 kg and 4 kg since early and middle-late adulthood respectively were associated with increased risks of NAFLD.

**Conclusion:**

Among current nonobese individuals, those with a history of obesity in their early adulthood had a higher risk of NAFLD than those never obese. Among the currently obese population, those who became obese after mid-adulthood have a significantly lower risk of NAFLD compared with those who were stable obese.

**Supplementary Information:**

The online version contains supplementary material available at 10.1186/s12967-023-04007-8.

## Background

Non-alcoholic fatty liver disease (NAFLD), the most common cause of chronic liver disease in both children and adults, is affecting approximately one-third of the global population [1]. Obesity is a widely recognized driving factor of NAFLD [2–4]. It causes fat accumulation in the liver and induces NAFLD. The concomitance of obesity such as insulin resistance (IR), metabolic syndrome, type 2 diabetes mellitus (T2DM), and elevated inflammatory cytokines could accelerate fat accumulation and liver damage [5]. In addition, obesity causes DNA methylation, which is involved in the prevalence and progression of NAFLD.[6].Obesity plays an important role in both the initial and progression of NAFLD. It was estimated that the prevalence of NAFLD in obese individuals ranges between 50 and 80% [5]. However, most previous studies mainly focus on the association between current weight status, ignoring the effect of historical weight status in NAFLD.

Former obesity has been proven to be associated with several poor outcomes. Evidence indicates that in the general population, non-obese individuals who were formerly obese have a higher risk of mortality than those never obese [7–10]. In addition, it has been proved that the duration of obesity is also associated with cardiac remodeling and increases the risk of cardiovascular disease and all-cause mortality [11–13]. Compared to an individual who has been obese for many years, an individual who just became obese might have a different risk for obesity-related disease. Obesity is closely associated with the presence of NAFLD, while the effect of the historical weight status and duration of obesity on NAFLD remains unknown.

Considering the lack of knowledge above, using national-wide U.S. population-based data from the third National Health and Nutrition Survey (NHANES III) database, we aimed to explore the association of former weight status with the presence of NAFLD.

## Methods

### Study design and participants

The NHANES is a program of studies designed and conducted by the National Center for Health Statistics (NCHS) and aims to assess the health and nutritional status of the non-institutionalized population in the US. NHANES III was conducted between 1988 and 1994. The study protocol was approved by the institutional review board of the NCHS. Written informed consent to participate in NHANES III was obtained from all participants.

This study is a cross-sectional study using NHANES III datasets. We included participants aged 40 years or over. The exposures of this study are weight change patterns across adulthood. The outcome is ultrasound-measured hepatic steatosis at baseline.

**Fig. 1:**
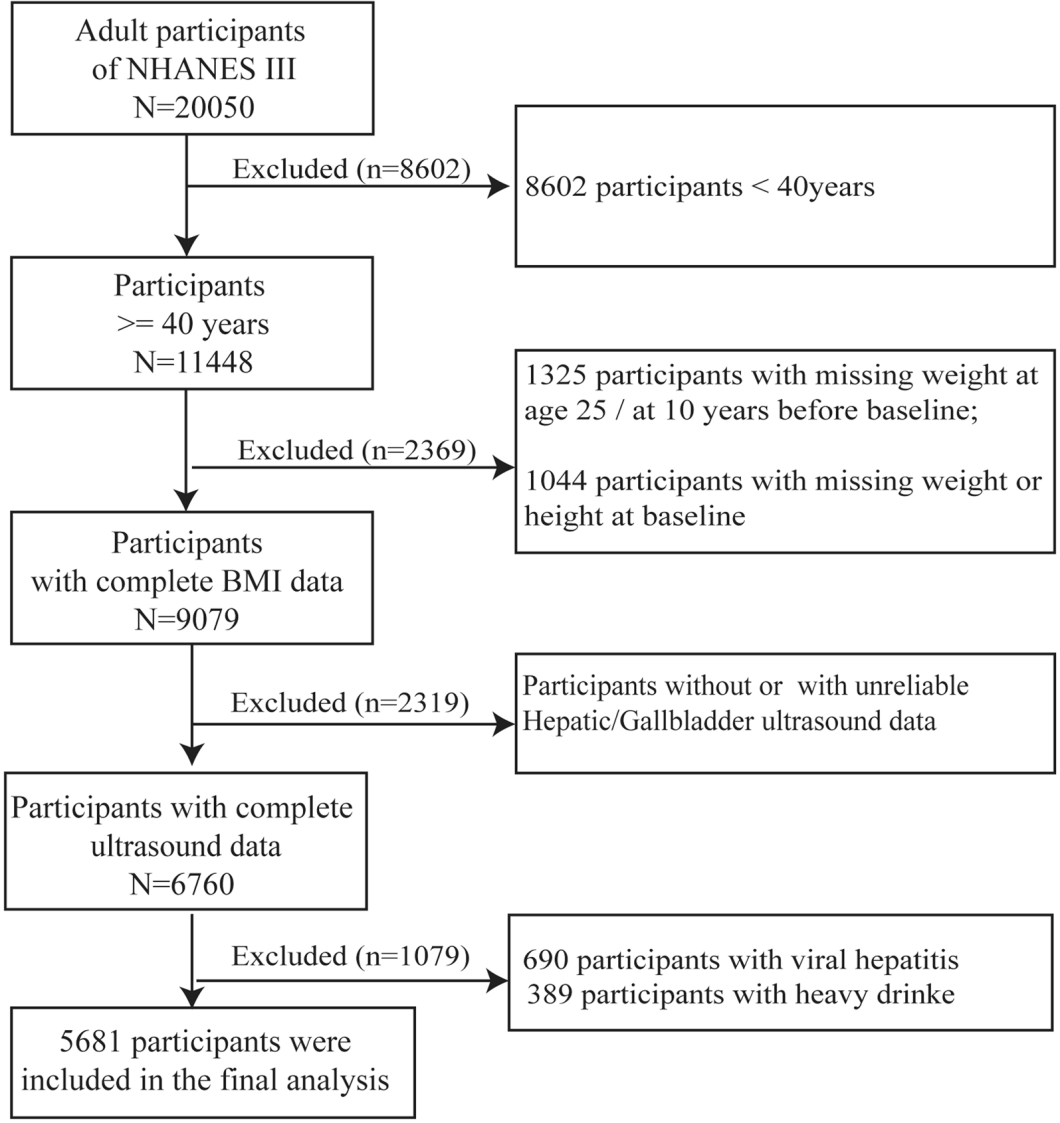
Participant Selection in National Health and Nutrition Examination Survey III. *NHANES* national health and nutrition examination Survey, *BMI* body mass index

Of the 20,050 adult participants from NHANES III, 8602 participants younger than 40 years were excluded, and 1325 participants with missing recalled weight data at age 25 years or 10 years before the survey were excluded. 1044 participants with missing baseline weight or height measurements were further excluded. We also excluded 2319 participants with missing or unreliable hepatic ultrasound data. 690 were further excluded due to viral hepatitis (positive serum hepatitis C antibody and/or positive serum hepatitis B surface antigen), and 389 were excluded due to excessive alcohol consumption (> 2 or 3 standard alcoholic drinks per day on average for women or men, respectively). The final study cohort included 5681 participants aged 40 years or older (Fig. 1).

### Assessments of BMI and weight change pattern

Body mass index (BMI) at age 25 years (BMI_25_), at 10 years before baseline survey (BMI_10prior_), and at baseline survey (BMI_baseline_) were calculated as recalled or survey-measured weight (kg) divided by the square of height (m^2^). Obesity was defined as BMI ≥ 30 kg/m^2^. According to the definition described previously [14]. We defined 4 weight change patterns in each of two intervals (early adulthood [age 25 to baseline survey], and middle and late adulthood [10 years before the survey to baseline survey]): stable non-obese (stay BMI < 30.0), obese to non-obese (BMI ≥ 30.0 to < 30.0), non-obese to obese (BMI < 30.0 to ≥ 30.0), and stable obesity (stay BMI ≥ 30.0). We also categorized absolute weight change into five groups: weight change < 2.5 kg (reference group), weight loss ≥ 2.5 kg, weight gain 2.5–9.9 kg, weight gain 10–19.9 kg, and weight gain ≥ 20.0 kg.

### Determinant of NAFLD

Detailed descriptions of methods used for the gallbladder/hepatic ultrasound-diagnosed fatty liver in NHANES III have been published elsewhere [15]. The hepatic steatosis was assessed by re-reviewing the archived gall bladder ultrasound video images between 2009 and 2010. The presence of fat within the hepatic parenchyma was evaluated by certified radiologists. In this study, NAFLD was diagnosed by having the presence of moderate or severe hepatic steatosis in the absence of excessive alcohol consumption and other causes of chronic liver disease.

### Covariates measurements

Sociodemographic characteristics [age, sex, race/ethnicity, family poverty income ratio (FIPR), marital status, health status] were self-report. Race/ethnicity was categorized into four groups (non-Hispanic white, Non-Hispanic Black, Mexican–American, and other). FIPR was calculated as total family income divided by the poverty threshold, categorized into three groups (< 1.3, 1.3 to 1.5, ≥ 3.5). Marital status was categorized into two groups (living with a partner, single), and health status was categorized into three groups (excellent or good, fair, or poor). Current smokers and diabetes were self-reported. Hypertension and hypercholesterolemia were both determined either self-reported or by the NHANES objective measurements [Systolic blood pressure ≥ 140 mmHg or diastolic blood pressure ≥ 90 mmHg for hypertension; total cholesterol (TC) ≥ 240 mg/dL for hypercholesterolemia]. Body measurements, including standing height (cm), weight (kg), waist circumference (cm), and blood pressure, were measured during the physical examination.

Blood tests included TC (mg/dL), triglycerides (TG) (mg/dL), low-density lipoprotein cholesterol (LDL-C) (mg/dL), high-density lipoprotein cholesterol (HDL-C) (mg/dL), Lipoprotein(a) [LP(a)] (mg/dL), C-reactive protein (CRP) (mg/dL), fasting glucose (mg/dL) and fasting insulin (uU/ml), alanine aminotransferase (ALT) (IU/L), aspartate aminotransferase (AST) (IU/L), γ-glutamyl transferase (GGT) (IU/L). Insulin resistance (IR) was assessed with the homeostatic model assessment–insulin resistance (HOMA-IR) equation as follows: fasting insulin (uU/mL) × fasting blood glucose (mmol/L) ÷ 22.5.

### Statistical analysis

All data are presented as mean (SD), or number (percentage), as appropriate. Baseline characteristics were compared using the chi-square test for categorical variables and unadjusted linear regressions for continuous variables. Age-standardized prevalence estimates were calculated for each weight change pattern subgroup. Logistic regression models were used to determine odds ratios (ORs) and corresponding 95% confidence intervals (CIs) for the associations between weight change patterns and the risk of NAFLD. We calculated population attributable fraction (PAF) to estimate the percentage of NAFLD cases that could be prevented under the following scenarios: [1] if those who were obese before the survey can lose weight and change to non-obese; [2] if those who changed from non-obese to obese before the survey to baseline can stay non-obese. Logistic regression models with restricted cubic splines (RCS) were applied to examine the odds of NAFLD and absolute weight changes. Statistical tests were 2-sided, and statistical significance was set at P < 0.05. All statistical analyses were conducted using R software, version 4.0.1 (R Core Team, Vienna, Austria).

## Results

### Baseline Characteristics according to weight change pattern

**Fig. 2:**
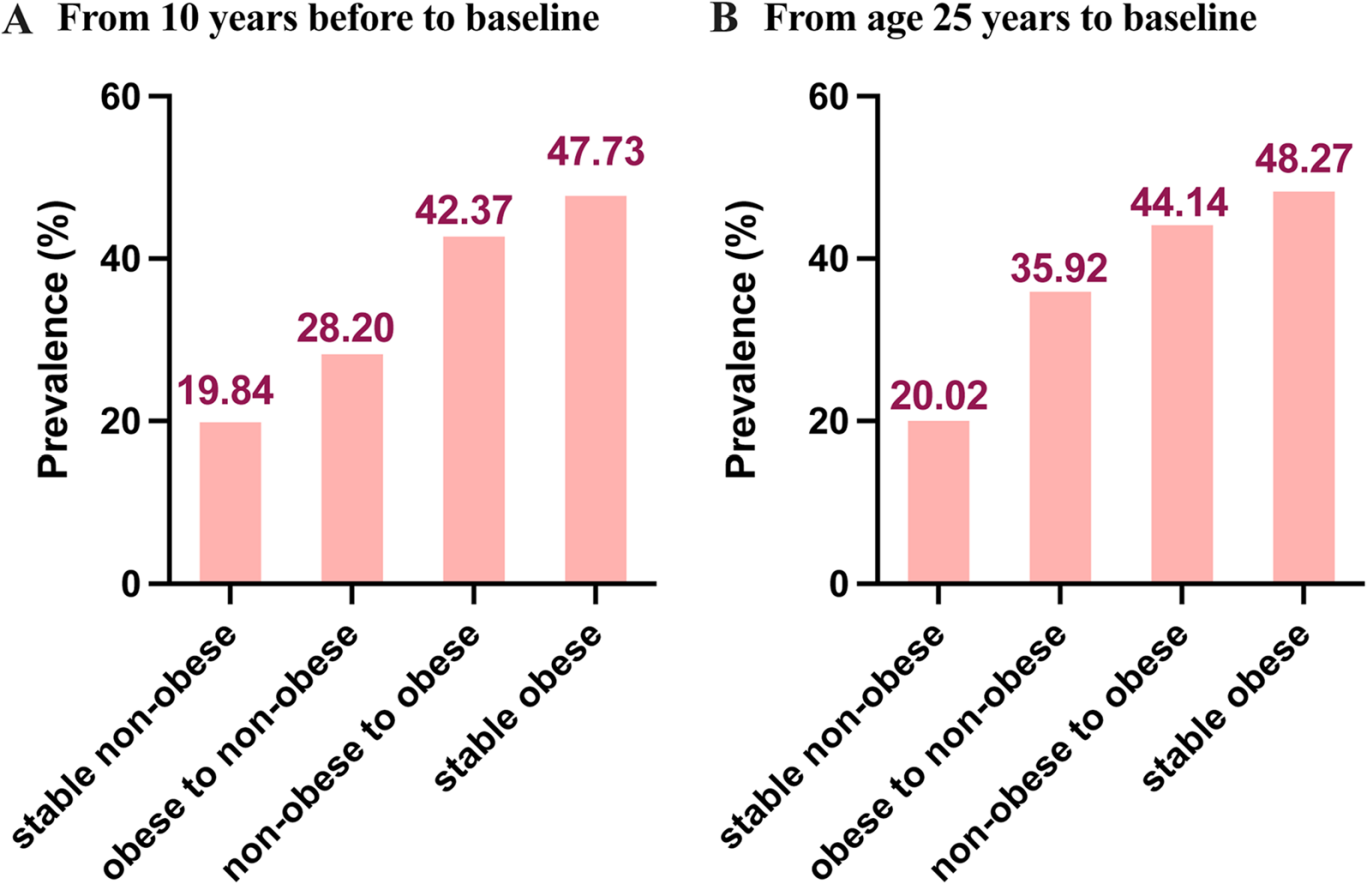
Adjusted NAFLD prevalence according to weight change patterns subgroups from age 25 years to baseline (**A**) and 10 years before the survey to baseline (**B**)

The study contained 5681 participants in the final analysis. From 10 years before the survey to baseline, 3681 were stable non-obese, 260 moved from obese to non-obese, 937 moved from non-obese to obese, 803 were stable obese. The age-adjusted NAFLD prevalence for these four groups were 19.84% (95% CI 18.43–21.34%), 28.20% (95% CI 21.63–36.48%), 42.37% (95% CI 38.12–47.03%) and 47.73% (95% CI 43.07–52.79%) respectively (Table 1 and Fig. 2 A). From age 25 to baseline, 3860 were stable non-obese, 81 moved from obese to non-obese, 1517 changed from non-obese to obese, 223 were stable obese. The age-adjusted NAFLD prevalence for these four groups were 20.02% (95% CI 18.63–21.48%), 35.92% (95% CI 21.63–60.42%), 44.14% (95% CI 40.82–47.69%) and 48.27% (95% CI, 39.08–59.78%) respectively (Additional file 1: Table S1 and Fig. 2B).

**Table 1:** Baseline characteristics of participants in NHANES III (1988–1994) according to weight change patterns from 10 years before the survey to baseline

	Stable nonobese	Obese to nonobese	P* value	Stable obese	Nonobese to obese	P^#^ value
	N = 3681	N = 260		N = 803	N = 937	
NAFLD	728 (19.8)	71 (27.3)	0.005	386 (48.1)	395 (42.2)	0.015
Age-adjusted NAFLD prevalence, %	19.84 (18.43–21.34)	28.20 (21.63–36.48)		47.73 (43.07–52.79)	42.37 (38.12–47.03)	
Age, years	56.14 (10.70)	60.13 (9.69)	< 0.001	56.80 (10.10)	53.77 (10.09)	< 0.001
Female	1873 (50.9)	116 (44.6)	0.059	483 (60.1)	581 (62.0)	0.458
Race/ethnicity						
Non-hispanic white	794 (21.6)	88 (33.8)	< 0.001	215 (26.8)	262 (28.0)	0.922
Non-Hispanic black	767 (20.8)	73 (28.1)		228 (28.4)	258 (27.5)	
Mexican–American	1983 (53.9)	97 (37.3)		339 (42.2)	390 (41.6)	
Other	137 (3.7)	2 (0.8)		21 (2.6)	27 (2.9)	
FIPR group						
< 1.3	717 (19.5)	90 (34.6)	< 0.001	213 (26.5)	255 (27.2)	0.013
1.3 to 3.5	1474 (40.0)	59 (22.7)		227 (28.3)	318 (33.9)	
≥ 3.5	1490 (40.5)	111 (42.7)		363 (45.2)	364 (38.8)	
Marital status						
Living with partner	2722 (74.0)	182 (70.5)	0.244	542 (67.8)	645 (69.1)	0.621
Single	954 (26.0)	76 (29.5)		257 (32.2)	289 (30.9)	
Health status						
Excellent or good	2842 (77.2)	141 (54.2)	< 0.001	494 (61.5)	664 (70.9)	< 0.001
Fair	692 (18.8)	81 (31.2)		231 (28.8)	227 (24.2)	
Poor	147 (4.0)	38 (14.6)		78 (9.7)	46 (4.9)	
Waist circumference, cm	90.54 (9.67)	98.01 (8.18)	< 0.001	114.60 (11.73)	107.79 (9.04)	< 0.001
BMI, kg/m2						
BMI_25_	22.03 (2.86)	25.30 (4.49)	< 0.001	27.28 (5.19)	23.45 (3.14)	< 0.001
BMI_10prior_	23.89 (2.88)	33.05 (3.93)	< 0.001	35.13 (4.84)	26.59 (2.40)	< 0.001
BMI_baseline_	24.97 (2.98)	27.57 (2.12)	< 0.001	36.47 (5.54)	33.19 (3.11)	< 0.001
Diabetes	343 (9.3)	96 (36.9)	< 0.001	272 (33.9)	148 (15.8)	< 0.001
Hypertension	1557 (42.3)	176 (67.7)	< 0.001	527 (65.6)	535 (57.1)	< 0.001
Hypercholesterolemia	1512 (41.7)	125 (48.8)	0.03	335 (42.4)	412 (44.9)	0.318
Current smoker	942 (25.6)	77 (29.6)	0.174	129 (16.1)	169 (18.0)	0.306
TC, mg/dL	218.14 (42.08)	220.62 (45.36)	0.368	219.47 (42.09)	223.19 (45.59)	0.086
TG, mg/dL	152.77 (118.83)	176.04 (113.99)	0.003	190.67 (146.32)	192.25 (141.29)	0.824
LDL-C, mg/dL	136.78 (37.77)	138.82 (40.32)	0.572	138.72 (37.19)	139.04 (39.79)	0.912
HDL-C, mg/dL	52.04 (16.30)	48.67 (16.78)	0.002	45.70 (12.76)	47.53 (14.24)	0.007
LP(a), mg/dL	25.84 (28.25)	29.58 (30.90)	0.151	27.29 (31.31)	25.52 (28.91)	0.366
CRP, mg/dL	0.21 [0.21, 0.40]	0.21 [0.21, 0.55]	0.002	0.44 [0.21, 0.90]	0.30 [0.21, 0.70]	< 0.001
HOMA-IR	1.86 [1.32, 2.84]	2.84 [1.83, 4.59]	< 0.001	4.02 [2.66, 7.29]	3.41 [2.34, 5.17]	< 0.001
Fast glucose, mg/dL	100.85 (35.48)	128.22 (73.87)	< 0.001	122.40 (59.46)	107.26 (41.79)	< 0.001
UA, mg/dL	5.25 (1.43)	5.58 (1.67)	0.001	5.83 (1.55)	5.79 (1.51)	0.565
Creatinine, mg/dL	1.10 (0.33)	1.28 (1.14)	< 0.001	1.10 (0.47)	1.08 (0.23)	0.331
AST, U/L	21.35 (10.87)	20.16 (8.07)	0.091	21.06 (9.69)	23.21 (15.24)	0.001
ALT, U/L	16.16 (12.17)	15.28 (8.90)	0.263	17.53 (10.36)	20.72 (15.71)	< 0.001
GGT, U/L	30.65 (36.11)	34.61 (34.64)	0.145	36.50 (37.42)	42.26 (66.60)	0.058

Data are given as mean ± SD, n (%), or median [interquartile range] as appropriate

^*^p-values were calculated for the comparison between stable non-obese and obese to non-obese NAFLD participants from age 25 years to baseline

^#^p-values were calculated for the comparison between non-obese to obese and stable obese NAFLD participants from age 25 years to baseline

*NHANES* national health and nutrition examination surveys, *CI* confidence interval, *FPIR* family poverty income ratio, *BMI* body mass index, *TC* total cholesterol, *TG* triglycerides, *LDL-C* low-density lipoprotein cholesterol, *HDL-C* high-density lipoprotein cholesterol, *LP(a)* Lipoprotein(a), *CRP* C-reactive protein, *HOMA-IR* homeostatic model assessment–insulin resistance, *UA* uric acid, *AST* aspartate aminotransferase, *ALT* alanine aminotransferase, *GGT* γ-glutamyl transferase

According to the weight change patterns from 10 years before the survey to baseline, compared with the stable non-obese group, participants in the obese to the non-obese group were older, had a larger waist circumference, were less likely to report excellent or good health status, more likely to have diabetes, performed worse lipid profile with higher LDL-C and lower HDL-C. Participants in the obese to the non-obese group also performed higher HOMA-IR and Fast glucose (Table 1). Compared with the non-obese to obese group, participants in the stable obese group were likely to be male and diabetes, had a larger waist circumference, performed higher CRP, HOMA-IR, and fast glucose, and there was no other significant difference in socioeconomic characteristics and laboratory parameters between the two groups. Detailed information showed in Table 1. Similar baseline characteristics were observed according to the weight change group from age 25 to baseline, (Additional file 1: Table S1). BMI of the study population showed a decreasing trend in the obese to the non-obese group over time and an increasing trend in the other three subgroups (Additional file 1: Figure S1.)

### Association between weight change pattern subgroups and NAFLD

Among current non-obese participants, those who were obese at age 25 years had a 1.82-fold increased risk of NAFLD (95% CI 1.17–2.92) than those who were never obese when adjusted for age, sex, race/ethnicity, and FIPR (Table 2). Those who were obese 10 years before the survey shared a similar risk of NAFLD with those stable non-obese (OR, 1.32; 95% CI, 0.98–1.76; Table 2). Among current obese participants, those who were non-obese at age 25 shared a similar risk of NAFLD with stable obese participants (OR, 0.82; 95% CI, 0.61–1.09). Those who were non-obese 10 years before the survey had a lower risk of NAFLD than stable obese participants (OR, 0.79; 95% CI, 0.65–0.96; Table 2).

**Table 2:** Association between weight change pattern subgroups and NAFLD

	Model 1		Model 2^a^	
	OR (95% CI)	P value	OR (95% CI)	P value
Age 25 to baseline				
Stable non-obese	1.00 (ref)		1.00 (ref)	
Obese to non-obese	2.00 (1.25–3.17)	0.004	1.82 (1.12–2.92)	0.014
Stable obese	1.00 (ref)		1.00 (ref)	
Non-obese to obese	0.81(0.61–1.08)	0.154	0.82 (0.61–1.09)	0.168
10 years prior to baseline				
Stable non-obese	1.00 (ref)		1.00 (ref)	
Obese to non-obese	1.52 (1.14–2.02)	0.003	1.32 (0.98–1.76)	0.066
Stable obese	1.00 (ref)		1.00 (ref)	
Non-obese to obese	0.78 (0.65–0.95)	0.135	0.79 (0.65–0.96)	0.018

^a^Adjusted for age, sex, race/ethnicity, and family poverty income ratio

*NAFLD* non-alcoholic fatty liver disease, *OR* odds ratio, *CI* confidence interval

PAFs for population counterfactuals are reported in Table 3. If those who were obese at age 25 years can lose weight and revert to non-obese, 25.84% (95% CI 6.62–44.42%) of observed NAFLD cases might have been averted. If those who were obese at 10 years before the survey can lose weight and change to non-obese, 38.30% (95% CI 26.83–49.76%) of observed NAFLD cases might have been averted. If those who were non-obese at age 25 can avoid becoming obese and stay non-obese, 22.92% (95% CI 19.79–26.01%) of observed NAFLD cases might have been averted. If those who were non-obese 10 years before the survey can stay non-obese, 17.27% (95% CI 14.29–20.26%) of observed NAFLD cases might have been averted.

**Table 3:** PAFs for population counterfactuals of NAFLD

	Scenario	Age 25 to baseline		10 years prior to baseline	
		PAF, %	95% CI	PAF, %	95% CI
Obese-non-obese vs stable obese	If those who were obese before the survey can lose weight and revert to non-obese	25.84	6.62, 45.42	38.30	26.83, 49.76
Stable non-obese vs non-obese to obese	If those non-obese before the survey avoid becoming obese and stay non-obese	22.92	19.79, 26.01	17.27	14.29, 20.26

*PAF* population attributable fraction, *NAFLD* non-alcoholic fatty liver disease, *CI* confidence interval

### Association of absolute weight change and NAFLD

**Fig. 3:**
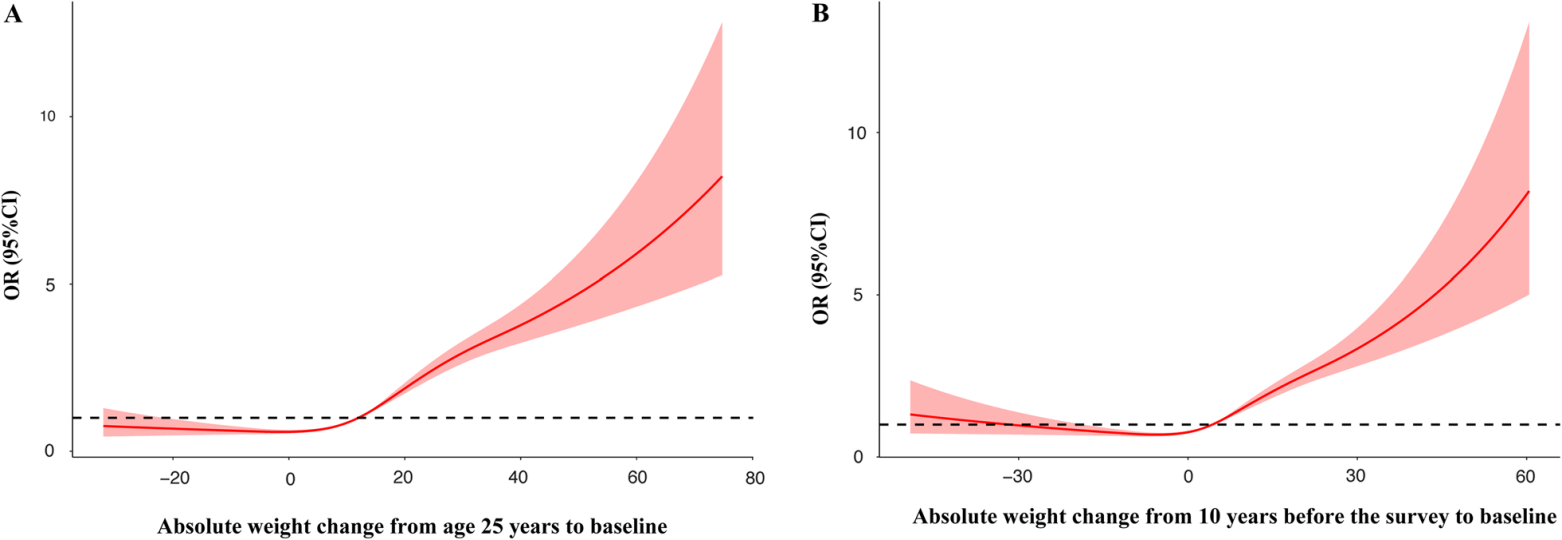
Dose–response association of the weight change from age 25 to baseline (**A**) and weight change from 10 years before the survey to baseline (**B**) with the prevalence of NAFLD. Restricted cubic splines were used with four knots. Odds ratios were indicated by solid lines and 95% CI by shaded areas. Covariates included age, sex, race/ethnicity, family poverty income ratio, hypercholesterolemia, hypertension, diabetes, and smoking. P for nonlinearity < 0.001

From age 25 to baseline, absolute weight change (per 1 kg increase OR, 1.03; 95%CI 1.03–1.04) was independently associated with the presence of NAFLD (Additional file 1: Table S2). Greater weight gain carried with it a successively higher risk of NAFLD, ORs rose steadily from 1.26 (95% CI 0.96–1.68) among those who gained weight within 2.5 to 10 kg, to 4.09 (95% CI 3.14–5.38) among those gained weight more than 20 kg (Additional file 1: Table S2). We further estimated the dose–response relationship between absolute weight change and NAFLD with RCS functions using 4 knots. A nonlinear association was observed (p < 0.001 for nonlinearity). The risk of the NAFLD was higher in participants with a weight gain at least of 12 kg (Fig. 3A).

From 10 years before the survey to baseline, weight change (per 1 kg increase OR: 1.03, 95%CI 1.03–1.04) was independently associated with the presence of NAFLD (Additional file 1: Table S2). Using the weight change within 2.5 kg as the reference, weight gain within 2.5 to 10 kg (OR, 1.66; 95% CI 1.38–2.00), weight gain within 10 to 20 kg, (OR, 2.47; 95% CI 2.02–3.03) and weight gain more than 20 kg (OR, 3.84; 95% CI 2.98–4.96) had a higher risk of the presence of NAFLD (Additional file 1: Table S2). Dose–response relationship between weight change from 10 years before the survey to baseline and NAFLD with RCS showed in Fig. 3B. A nonlinear association was observed (p < 0.001 for nonlinearity), and the risk of NAFLD was higher in participants with a weight gain at least of 4.0 kg (Fig. 3B).

## Discussion

The main results of the present study were the followings: (1) Both current and former obesity status were associated with the presence of NAFLD. (2) Among current nonobese individuals, those with a history of obesity in their early adulthood had a higher risk of NAFLD than those never obese. (3) Among the currently obese population, those who became obese after mid-adulthood have a significantly lower risk of NAFLD compared with those who were stable obese. 4) A weight gain of more than 12 kg and 4 kg since early and middle-late adulthood respectively were associated with increased risks of NAFLD. 5) If those who were obese formerly can lose weight and revert to non-obese, as well as those who were non-obese can avoid becoming obese, observed NAFLD cases might have been significantly averted.

The pathophysiology of the relationship between obesity and NAFLD was mainly mediated by adipose tissue dysfunction and hepatic de-novo lipogenesis. Obesity is closely correlated with the expansion of adipose tissue, which deteriorates its capability in storing excess energy, inducing adipocyte dysfunction [16]. In the context of adipose dysfunction, macrophages infiltrate into the adipose tissue and induce inflammation that promotes IR [17]. As adipocyte dysfunction and IR increases, lipolysis resulting elevated circulating free fatty acids (FFA), which then becomes available for uptake by the liver and overwhelms its metabolic capacity [18]. These pathophysiological changes resulting in intrahepatic fat accumulation, which is a prominent characteristic of steatosis. In obese individuals, it is commonly observed that steatosis is further amplified by excessive dietary fat and carbohydrate intake, which also increases de novo lipogenesis [5]. Besides, obesity also affects gene expression through numerous epigenetic mechanisms such as DNA methylation, which is involved in the prevalence and progression of NAFLD [6]. A number of studies have demonstrated altered DNA methylation profiles such as the methylation levels of peroxisome proliferator-activated receptor-gamma coactivator 1a [19] and mitochondria-encoded NADH dehydrogenase in liver biopsy samples collected from NAFLD patients are important in the development of NAFLD [20]. Although abundant evidence has demonstrated the mechanism of the relationship between current obesity and NAFLD, few studies focused on the effect of historical weight status.

It has been well recognized that historical and current weight status and absolute weight change over time were all associated with various metabolic-related diseases including cardiometabolic disease, heart failure, diabetes, and hypertension [8–10, 21–25]. Several clinical trials suggest that intentional weight loss was associated with remission of NAFLD both in obese and non-obese patients [26–30]. However, little is known whether historical weight change in various pattern is associated with NAFLD in the real-world scenarios. The current study found that former obesity is a risk factor for the presence of NAFLD. Compared with never-obese participants, those who were obese formerly in their adulthood had a higher risk of NAFLD. These findings are consistent with the previous study focused on weight status and all-cause mortality, which claimed that compared to those never obese, participants who were formerly obese had a higher mortality risk [7–10, 14]. Our results imply that the risk of obesity on NAFLD cannot be completely eliminated by weight loss and highlight the importance of weight maintenance throughout the lifetime.

The study also adds evidence that the risk of obesity on NAFLD increases with the duration of obesity. Several characteristics of obesity, i.e. the obesity timing and the rate of being obese, affect NAFLD. Body weight gain during earlier adulthood was more strongly associated with NAFLD than those during later adulthood, and body weight gain rate further adds risk for NAFLD. [31–33]. It is reasonable to hypothesize that the duration, another characteristic of obesity, also plays an important role in the development of NAFLD. The risk for NAFLD of a patient who just became obese might be different from another who has been obese for the past 20 years. The association between obesity duration and cardiovascular disease was fully studied [12–14]. Nakajima et al. [12] found that alterations of cardiac performance in obese patients with left ventricular enlargement and wall thickening are attributed not only to the excess of body weight but also to the duration of obesity. Abdullah et al. [13] included 5036 participants of the Framingham Cohort Study and claimed that the risk of all-cause mortality increased as the number of years lived with obesity increased independent of a set of potential confounders and even current BMI. Chen et al. [14] found that those who were stable obese with longer obesity duration performed at higher risk of all-cause and heart disease mortality. In our results, the longer duration of obesity is associated with a higher risk of NAFLD. The result highlights that public health policies aiming to prevent obesity should start as early as possible.

In this study, there are statistically significant differences in ages between weight change pattern subgroups. Previous studies indicated that the prevalence and incidence of NAFLD increased with age in a stepwise manner [34, 35]. It can be explained that with age increasing, subcutaneous adipose tissue dysfunction would be induced via the accumulation of senescent adipocytes, impaired preadipocyte development, and reduced mitochondrial activity which leads to the incidence of NAFLD [36–38]. According to the age-adjusted prevalence and results of the multivariable analysis, our findings suggest that the effect of weight change patterns on NALFD is independent of age.

The strengths of our study include its large and nationally representative sample, and high follow-up rate, our study highlights that it is important to avoid obesity throughout overall adulthood and the public health policies aiming to prevent obesity should start as early in age as possible. Several limitations of this study should also be considered. First, recalled and self-reported weight data before the survey may exist bias. Second, participants with missing recalled weight data were excluded from the present study, thus, participants in this study may not represent the general population in the real world. Third, we were unable to further ascertain the relationship between formerly obesity and the duration of obesity with liver-related mortality data since the NCHS restricts this information for public usage. Finally, historical weight data were only collected at 2-time points [25 years old and 10 years before baseline] with no extra data on body weight fluctuations during these time intervals in NHANES III. Further research is needed to evaluate the relationship between dynamic weight fluctuations and NAFLD. Nevertheless, we believe that these limitations can be offset by the benefits of a large population-based study with the use of a nationally representative sample, the long duration of the follow-up period, and the generalizability of our results to an ethnically diverse Western population in the US.

## Conclusion

In conclusion, the prevalence of NAFLD varied by both current and former weight status. The risk of obesity on NAFLD cannot be completely eliminated by weight loss, and the longer the period of obesity, the higher the risk of NAFLD.

## Supplementary Information

**Additional file 1: Table S1.** Baseline characteristics of participants in NHANES III (1988-1994) according to weight change patterns from age 25 years to baseline. **Table S2.** Association between absolute weight change subgroups and NAFLD. **Figure S1.** BMI trends over time from age 25 years to baseline according to weight change pattern subgroups.

## Data Availability

The National Health and Nutrition Examination Survey dataset is publicly available at the National Center for Health Statistics of the Center for Disease Control and Prevention (https://wwwn.cdc.gov/nchs/nhanes/nhanes3/datafiles.aspx).

## Abbreviations

NAFLD: Non-alcoholic fatty liver disease
BMI: Body mass index
CI: Confidence interval
OR: Odds ratio
NCH: National center for health statistics
NHANES III: The third national health and nutrition survey
FIPR: Family poverty income ratio
TC: Total cholesterol
TG: Triglycerides
LDL-C: Low-density lipoprotein cholesterol
HDL-C: High-density lipoprotein cholesterol
LP(a): Lipoprotein(a)
CRP: C-reactive protein
ALT: Alanine aminotransferase
AST: Aspartate aminotransferase
GGT: γ-Glutamyl transferase
IR: Insulin resistance
HOMA-IR: Homeostatic model assessment–insulin resistance
PAF: Population attributable fraction
RCS: Restricted cubic splines
