# Supplementary material for: Weigh change across adulthood is related to the presence of NAFLD: results from NHANES III
Source: J Transl Med. 2023 Feb 23;21:142. doi: 10.1186/s12967-023-04007-8 (PMC9951528; doi:10.1186/s12967-023-04007-8)
Supplement: Supplementary file 1 — Additional file 1: Table S1. Baseline characteristics of participants in NHANES III (1988-1994) according to weight change patterns from age 25 years to baseline. Table S2. Association between absolute weight change subgroups and NAFLD. Figure S1. BMI trends over time from age 25 years to baseline according to weight change pattern subgroups. [file 12967_2023_4007_MOESM1_ESM.docx]

**Weigh change across adulthood is related to the presence of NAFLD: results from NHANES III**

Lili Wang ^2*^, Jiayi Yi ^2*^, Jiajun Guo ^3^, Xiangpeng Ren ^1†^

* Lili Wang and Jiayi Yi contributed equally as co-first authors for this article

† Corresponding author

**Affiliation**

^1^ Department of Biochemistry, Medical College, Jiaxing University, Jiaxing, Zhejiang Province, 314001, China

^2^ Department of Cardiology, Fuwai Hospital, Chinese Academy of Medical Sciences and Peking Union Medical College, National Center for Cardiovascular Diseases, Beijing, 100035, China.

^3^ Department of Cardiology, West China Hospital, Sichuan University, Chengdu, Sichuan Province, 610041, China.

**Contact Info**

Xiangpeng Ren, Department of Biochemistry, Medical College, Jiaxing University, Jiaxing, China. No.899 Guangqiong Road, Jiaxing City, Zhejiang Province, 314001, China.

Telephone: +86-13738362415.

E-mail: renxpeng@mail3.sysu.edu.cn

Telephone: +86-15810911931.

**Additional file 1: Table S1. Baseline characteristics of participants in NHANES III (1988-1994) according to weight change patterns from age 25 years to baseline.**

|  | **Non-obese at baseline survey** | | |  | **Obese at baseline survey** | | |
| --- | --- | --- | --- | --- | --- | --- | --- |
|  | Stable non-obese | Obese to  non-obese | P^*^ value |  | Stable obese | Non-obese to obese | P^#^ value |
|  | (N=3860) | (N=81) |  |  | (N=223) | (N=1517) |  |
| **NAFLD** | 772 (20.0) | 27 (33.3) | 0.005 |  | 110 (49.3) | 671 (44.2) | 0.175 |
| **Age-adjusted NAFLD prevalence, %** | 20.02  (18.63-21.48) | 35.92  (21.63-60.42) |  |  | 48.27  (39.08-59.78) | 44.14  (40.82-47.69) |  |
| **Age, years** | 56.35 (10.69) | 59.04 (10.23) | 0.025 |  | 54.03 (10.75) | 55.34 (10.11) | 0.073 |
| **Female,** | 1954 (50.6) | 35 (43.2) | 0.227 |  | 121 (54.3) | 943 (62.2) | 0.029 |
| **Race/ethnicity,** |  |  |  |  |  |  |  |
| Non-Hispanic White | 859 (22.3) | 23 (28.4) | 0.214 |  | 47 (21.1) | 430 (28.3) | 0.049 |
| Non-Hispanic Black | 819 (21.2) | 21 (25.9) |  |  | 68 (30.5) | 418 (27.6) |  |
| Mexican-American | 2044 (53.0) | 36 (44.4) |  |  | 105 (47.1) | 624 (41.1) |  |
| Other | 138 (3.6) | 1 (1.2) |  |  | 3 (1.3) | 45 (3.0) |  |
| **FPIR group,** |  |  |  |  |  |  |  |
| <1.3 | 782 (20.3) | 25 (30.9) | 0.004 |  | 53 (23.8) | 415 (27.4) | 0.009 |
| 1.3 to 3.5 | 1515 (39.2) | 18 (22.2) |  |  | 56 (25.1) | 489 (32.2) |  |
| ≥3.5 | 1563 (40.5) | 38 (46.9) |  |  | 114 (51.1) | 613 (40.4) |  |
| **Marital status,** |  |  |  |  |  |  |  |
| Living with partner | 2845 (73.8) | 59 (72.8) | 0.94 |  | 148 (67.3) | 1039 (68.7) | 0.734 |
| Single | 1008 (26.2) | 22 (27.2) |  |  | 72 (32.7) | 474 (31.3) |  |
| **Health status,** |  |  |  |  |  |  |  |
| Excellent or good | 2937 (76.1) | 46 (56.8) | <0.001 |  | 144 (64.6) | 1014 (66.8) | 0.774 |
| Fair | 752 (19.5) | 21 (25.9) |  |  | 63 (28.3) | 395 (26.0) |  |
| Poor | 171 (4.4) | 14 (17.3) |  |  | 16 (7.2) | 108 (7.1) |  |
| **Waist circumference, cm** | 90.93 (9.75) | 96.14 (8.33) | <0.001 |  | 118.22 (13.32) | 109.83 (10.07) | <0.001 |
| **BMI, kg/m2** |  |  |  |  |  |  |  |
| BMI_25_ | 22.03 (2.73) | 32.44 (2.48) | <0.001 |  | 34.07 (4.17) | 23.92 (2.96) | <0.001 |
| BMI_10prior_ | 24.37 (3.57) | 30.62 (5.76) | <0.001 |  | 36.76 (6.43) | 29.61 (4.91) | <0.001 |
| BMI_baseline_ | 25.11 (3.00) | 26.69 (2.63) | <0.001 |  | 38.02 (6.58) | 34.21 (4.13) | <0.001 |
| **Diabetes** | 413 (10.7) | 26 (32.1) | <0.001 |  | 69 (30.9) | 351 (23.1) | 0.014 |
| **Hypertension** | 1691 (43.8) | 42 (51.9) | 0.183 |  | 146 (65.5) | 916 (60.4) | 0.167 |
| **Hypercholesterolemia** | 1598 (42.0) | 39 (48.1) | 0.321 |  | 91 (41.4) | 656 (44.1) | 0.487 |
| **Current smoker** | 992 (25.7) | 27 (33.3) | 0.154 |  | 42 (18.8) | 256 (16.9) | 0.529 |
| **TC, mg/dL** | 218.28 (42.30) | 219.19 (42.68) | 0.85 |  | 218.71 (39.94) | 221.87 (44.60) | 0.329 |
| **TG, mg/dL** | 153.76 (118.25) | 180.05 (133.95) | 0.051 |  | 194.16 (207.59) | 191.13 (131.74) | 0.774 |
| **LDL-C, mg/dL** | 136.68 (38.00) | 151.18 (32.74) | 0.045 |  | 141.70 (33.67) | 138.44 (39.31) | 0.439 |
| **HDL-C, mg/dL** | 51.93 (16.39) | 46.68 (13.65) | 0.005 |  | 45.42 (13.36) | 46.86 (13.63) | 0.154 |
| **LP(a), mg/dL** | 26.04 (28.48) | 28.17 (27.48) | 0.609 |  | 25.70 (30.64) | 26.45 (30.00) | 0.8 |
| **CRP, mg/dL** | 0.21 [0.21, 0.40] | 0.21 [0.21, 0.38] | 0.891 |  | 0.44 [0.21, 0.99] | 0.33 [0.21, 0.77] | 0.002 |
| **HOMA-IR** | 1.91 [1.34, 2.96] | 2.31 [1.59, 3.60] | 0.012 |  | 4.10 [2.73, 7.21] | 3.58 [2.47, 5.93] | 0.017 |
| **Fast glucose, mg/dL** | 102.33 (39.00) | 118.13 (64.75) | 0.001 |  | 123.87 (68.70) | 112.86 (48.07) | 0.004 |
| **UA, mg/dL** | 5.27 (1.44) | 5.42 (1.70) | 0.37 |  | 5.98 (1.53) | 5.79 (1.53) | 0.092 |
| **Creatinine, mg/dL** | 1.11 (0.43) | 1.19 (0.61) | 0.107 |  | 1.10 (0.23) | 1.09 (0.38) | 0.695 |
| **AST, U/L** | 21.31 (10.79) | 19.47 (5.50) | 0.136 |  | 21.54 (9.12) | 22.31 (13.48) | 0.422 |
| **ALT, U/L** | 16.13 (12.06) | 14.82 (7.16) | 0.341 |  | 18.21 (11.66) | 19.39 (13.85) | 0.241 |
| **GGT, U/L** | 31.00 (36.32) | 26.77 (19.02) | 0.352 |  | 32.15 (22.73) | 40.63 (58.01) | 0.061 |

Data are given as mean ± SD, n (%), or median [interquartile range) as appropriate.

^*^ p-values were calculated for the comparison between stable non-obese and obese to non-obese NAFLD participants from age 25 years to baseline.

^#^ p-values were calculated for the comparison between non-obese to obese and stable obese NAFLD participants from age 25 years to baseline.

Abbreviations: NHANES, National Health and Nutrition Examination Surveys; CI, confidence interval; FPIR, family poverty income ratio; BMI, body mass index; TC, total cholesterol; TG, triglycerides; LDL-C, low-density lipoprotein cholesterol; HDL-C, high-density lipoprotein cholesterol; LP(a), Lipoprotein(a); CRP, C-reactive protein; HOMA-IR, homeostatic model assessment–insulin resistance; UA, uric acid; AST, aspartate aminotransferase; ALT, alanine aminotransferase; GGT, γ-glutamyl transferase.

Additional file 1: **Table S2. Association between absolute weight change subgroups and NAFLD.**

|  | Number of NAFLD /total | **Model 1** | |  | **Model 2** | |
| --- | --- | --- | --- | --- | --- | --- |
|  |  | **OR (95% CI)** | **P value** |  | **OR (95% CI)** | **P value** |
| **Age 25 to baseline** |  |  |  |  |  |  |
| Absolutely weight change |  | 1.04 | <0.001 |  | 1.03 | <0.001 |
|  |  |  |  |  |  |  |
| Weight change within 2.5 kg | 84 / 529 | 1.00 (ref) |  |  | 1.00 (ref) |  |
| Weight loss ≥ 2.5 kg | 90 / 559 | 0.98 (0.71-1.37) | 0.919 |  | 0.92 (0.66-1.29) | 0.620 |
| Weight gain ≥ 2.5 and <10 | 251 / 1330 | 1.26 (0.96-1.67) | 0.094 |  | 1.26 (0.96-1.68) | 0.102 |
| Weight gain ≥10 and <20 kg | 476 / 1701 | 2.14 (1.66-2.79) | <0.001 |  | 2.02 (1.56-2.66) | <0.001 |
| Weight gain ≥20 kg | 679 / 1562 | 4.66 (3.61-6.07) | <0.001 |  | 4.09 (3.14-5.38) | <0.001 |
|  |  |  |  |  |  |  |
| **10 years prior to baseline** |  |  |  |  |  |  |
| Absolutely weight change |  | 1.04 (1.03-1.04) | <0.001 |  | 1.03 (1.03-1.04) | <0.001 |
|  |  |  |  |  |  |  |
| Weight change within 2.5 kg | 250 / 1252 | 1.00 (ref) |  |  | 1.00 (ref) |  |
| Weight loss > 2.5 kg | 292 / 1215 | 1.22 (1.01-1.49) | 0.042 |  | 1.01 (0.82-1.24) | 0.918 |
| Weight gain, >2.5-10 | 456 / 1672 | 1.67 (1.39-2.00) | <0.001 |  | 1.66 (1.38-2.00) | <0.001 |
| Weight gain, 10-20 kg | 370 / 1070 | 2.58 (2.12-3.14) | <0.001 |  | 2.47 (2.02-3.03) | <0.001 |
| Weight gain >20 kg | 212 / 472 | 4.54 (3.55-5.80) | <0.001 |  | 3.84 (2.98-4.96) | <0.001 |

Abbreviations: NAFLD, Non-alcoholic fatty liver disease; OR, odds ratio; CI, confidence interval. Model 1, adjusted for age, sex, race/ethnicity, and family poverty income ratio. Model 2, additionally adjusted for waist circumference, hypercholesterolemia, hypertension, diabetes, and smoking.

Additional file 1: **Figure S1. BMI trends over time from age 25 years to baseline according to weight change pattern subgroups.**

**
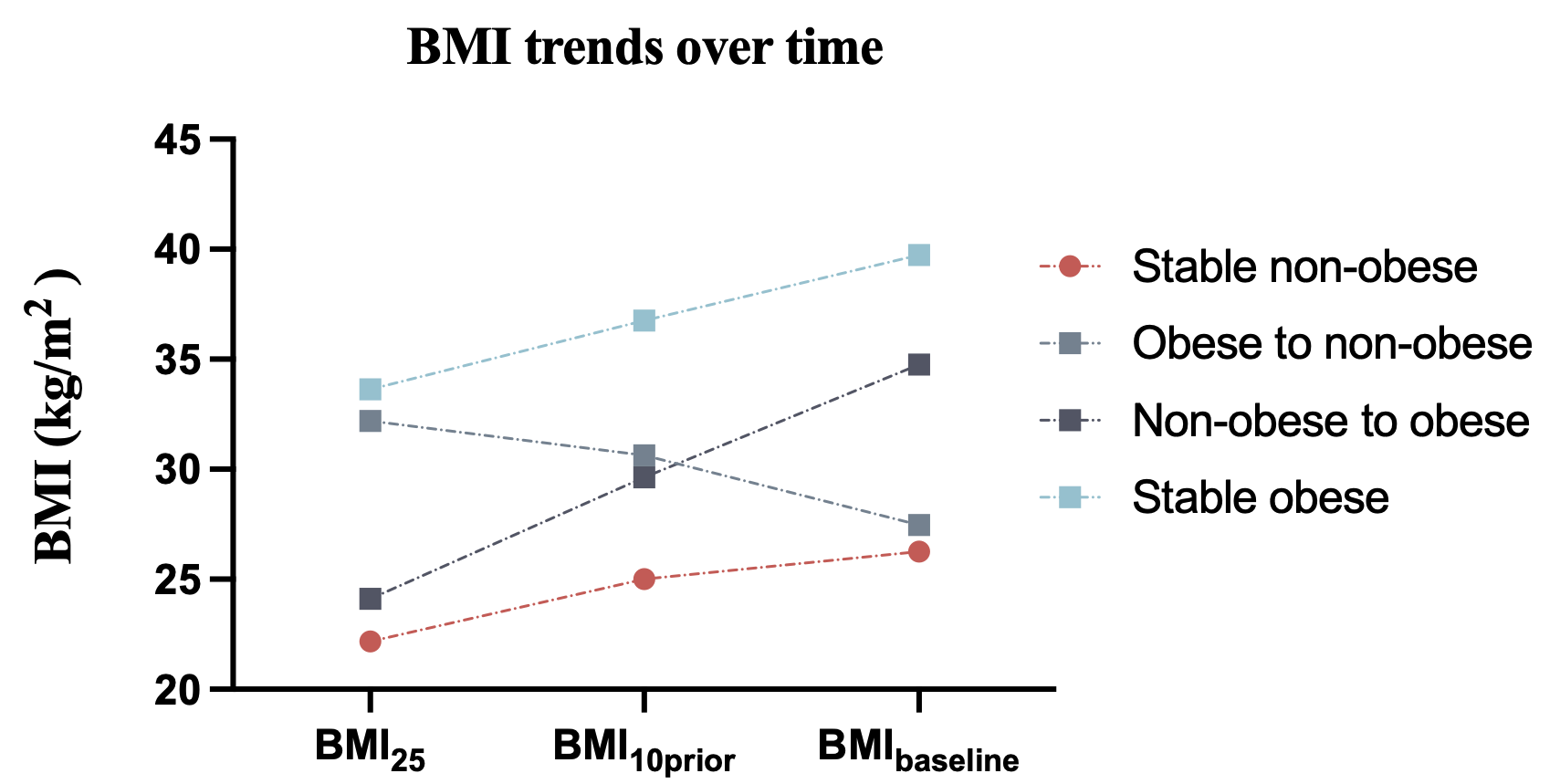
**
